# Twenty-seven continental ancestry-informative SNP analysis of bone remains to resolve a forensic case

**DOI:** 10.1080/20961790.2017.1306431

**Published:** 2017-05-19

**Authors:** Qifan Sun, Li Jiang, Guangfeng Zhang, Jing Liu, Lei Zhao, Wenting Zhao, Caixia Li

**Affiliations:** Key Laboratory of Forensic Genetics, Beijing Engineering Research Center of Crime Scene Evidence Examination, Institute of Forensic Science, Ministry of Public Security, Beijing, China

**Keywords:** Forensic science, skeletal remains, ancestry inference, SNPs, multiplex assay, capillary electrophoresis

## Abstract

We employed our previously developed 27-plex ancestry-informative single nucleotide polymorphism (SNP) panel to infer the ancestral components of bone remains of a possible foreign pilot found in south-western China. For ancestry assignment of this unknown individual, we first obtained the 27-SNP genotype of the individual. Then, based on a reference database of 3081 individuals from 33 populations, we calculated the match probability and likelihood ratio using the self-developed software program Forensic Intelligence. Inferred ancestral components of this individual were calculated by structure at *K* = 3. A complete profile was obtained for the individual using our multiplexed SNP assay. The European population was within one order of magnitude of the highest likelihood. The major ancestral component of this individual was 97.6% European.

## Introduction

Different genetic and physical characteristics arose during long-term human migration and evolution, yielding a large number of genetic markers with frequency variations in the human genome [1]. By detecting a certain set of such genetic markers, one can trace the population origin of an unknown DNA sample or infer the possible physical characteristics of the DNA contributor [2–4]. Due to their large number and wide distribution in the human genome, single nucleotide polymorphisms (SNPs) have become one of the most commonly used genetic markers for ancestry inference. Numerous ancestry-informative SNP (AISNP) panels have been published. Globally, ancestry resolution varies from three to eight groups of populations [5,6]. Three-continent resolution is also possible with relatively few AISNPs [7].

We previously developed a 27-plex SNP panel, consisting of a 27-plex SNP amplification system that utilizes capillary electrophoresis and a self-developed match probability (MP) calculation software (Forensic Intelligence version 1.0) [8,9]. Using this system, we can infer the ancestry origin of an unknown DNA sample at the three-continent level: African, European and East Asian. This multiplex assay is useful in criminal investigations where it can provide crucial investigative leads in a case when there is no database or suspect match.

In this paper, we demonstrate the method for ancestral component analysis of unknown bone remains found in south-western China.

## Case background

In August 2015, skeletal remains and aircraft wreckage found on a snow-covered mountain in south-western China were assumed to be linked to a foreign pilot who crashed during the Second World War. However, ancestry inference had to be performed before the skeletal remains could be returned to the pilot's country of origin. In this particular case, traditional forensic anthropology methods could not be applied to identify the ancestry of the remains because the skeleton was incomplete and poorly preserved. Thus, two pieces of femur were sent to our laboratory for DNA-based ancestry inference. We performed the analysis using our 27-plex AISNP panel.

## Materials and methods

### DNA sample preparation

DNA from the femurs was extracted using a previously developed method in our laboratory, i.e. cetyltrimethylammonium bromide lysis and isoamyl alcohol-chloroform extraction with subsequent DNA purification using the QIA quick system [10]. Then the DNA was quantified using a Nano Drop 2000c Spectrophotometer (Thermo Fisher Scientific, Wilmington, DE, USA). In total, 20 μL of extracted DNA was obtained from each of the femur pieces, with DNA concentrations of 60 and 58 ng/μL, respectively. DNAs were diluted to a concentration of 0.1 ng/μL for short tandem repeat (STR) tests and 5 ng/μL for 27-plex SNP assay analysis. A total of 5 ng of DNA was used in the experiment.

### Experimental method

Autosomal STR analysis was performed using the PowerPlex® 21 kit (Promega, Madison, WI, USA) and a GeneAmp 9700 Thermal Cycler (Applied Biosystems, Foster City, CA, USA). Each reaction contained 5 μL of DNA/sterile distilled water, 5 μL of PowerPlex® 21 5× Master Mix, 5 μL of PowerPlex® 21 5× Primer Pair Mix, and 10 μL water (Amplification Grade). Thermal cycling was performed as described by the manufacturer. AmpFLSTR Control DNA 9947A (Applied Biosystems, 0.1 ng/μL) was used as a positive control, and no DNA template was used as a negative control. Y-STR testing was performed using the PowerPlex® Y23 kit (Promega) and a GeneAmp 9700 Thermal Cycler (Applied Biosystems). Each reaction contained 5 μL of DNA/sterile distilled water, 5 μL of PowerPlex® 21 5× Master Mix, 2.5 μL of PowerPlex® 21 10× Primer Pair Mix, and 12.5 μL of water (Amplification Grade). Thermal cycling was performed as described by the manufacturer. AmpFLSTR Control DNA 2800M (Applied Biosystems, 0.1 ng/μL) was used as a positive control, and no DNA template was used as a negative control.

Samples were genotyped with our previously developed 27-plex SNP assay [8]. A one-tube 27-plex PCR followed by a 27-plex single-base extension reaction (SNaPshot®, Life Technologies, Wilmington, DE, USA) was performed, and the products were detected on a 3130xl capillary electrophoresis analyser (Life Technologies). Three positive DNA controls (European, African, and East Asian controls) were applied to every assay. One negative control (no DNA template) was processed using the same reaction reagents. Both of the bone DNA extractions were tested in parallel, and three replicates were performed for each.

### Data analysis

A reference database of 3081 individuals from 33 populations (Supplemental Table 1) was employed to calculate the population MP and inferred ancestral components. MP was calculated using the self-developed Forensic Intelligence v1.0 software (FI version 1.0, available online: https://github.com/jiangl1989/FI/) [8,11].The likelihood ratio was calculated based on AMP (assignment match probability) as described in previous reports [5,12]. Inferred ancestral components were calculated using STRUCTURE v2.3.4 software (https://web.stanford.edu/group/pritchardlab/structure.html) at at *K* = 3, using 10000 burn-ins, 10000 repetitions; admixture model, and an admixture model.

## Results

Autosomal STR and Y-STR results confirmed that the two pieces of femur belong to one person (data not shown). All of the replicates of the SNP assay generated the same SNP genotype. Supplemental Figure 1 displays a complete electropherogram profile obtained using our multiplexed SNP assay. The SNP genotyping for the sample is shown in Table 1.

**Figure 1.**
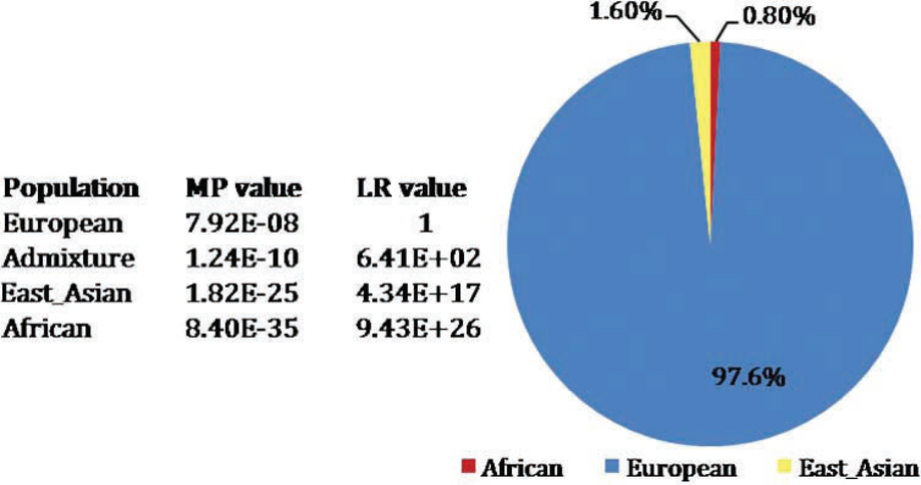
Results of the STRUCTURE components analysis) match probability (MP) and likelihood ratio (LR) calculations for the individual.

**Table 1.** SNP genotyping for the unknown bone sample.

Number	SNPs	Alleles
1	rs595961	AA
2	rs2710684	CC
3	rs260690	AA
4	rs10496971	TT
5	rs10497191	CT
6	rs820371	CT
7	rs1586861	TT
8	rs28777	AA
9	rs10079352	CT
10	rs1366220	CT
11	rs6875659	GG
12	rs7752055	TT
13	rs10258063	GG
14	rs366178	GT
15	rs4749305	AG
16	rs4244304	CT
17	rs10741584	AA
18	rs3825663	TT
19	rs728404	GG
20	rs1448485	CC
21	rs7170869	AA
22	rs1453858	AT
23	rs2470102	AA
24	rs4787040	AT
25	rs881929	TT
26	rs1197062	AA
27	rs4789193	TT

Results for the ancestry analysis of the pilot's skeletal remains are shown in Figure 1. The major ancestral component was 97.6% European. All four European populations were within one order of magnitude of the highest likelihood, with a MP of 7.92E-08 for European, 1.24E-10 for Admixture (Admixture of European and East Asian), 1.82E-25 for East Asian and 8.40E-35 for African. The results indicated that the bone remains likely belonged to a European individual.

## Discussion

In forensic applications, small sets of AISNPs have been shown to be ideal to meet the needs for trace DNA detection. It is relatively easy to build a small AISNP panel for three-continent population inferences [13]. Our single-tube 27-SNP panel works efficiently using 200 pg of DNA [8]. The system can also be successfully used to test commonly found forensic exhibits, such as blood stains, semen stains, touch DNA, bones and teeth (data not shown). The bone remains assayed here were recovered after more than 60 years of exposure to poor environmental conditions, but a complete genotype profile was still successfully obtained.

The 27-SNP panel includes eight African, nine European and 10 East Asian AIMs, providing nearly balanced discrimination power for each of the three ancestries (average Fst = 0.696, 0.750, and 0.673 between African and European, African and East Asian, and European and East Asian, respectively) [8]. This system was validated using more than 2000 individuals, demonstrating that it is robust for ancestry inference of individuals with African, European, and East Asian ancestry, as well as Eurasian admixture (EUR/EAS) [9]. The combined usage of ancestral components and likelihood ratios facilitates the ancestry inference of an unknown individual.

Aside from the specific AIMs selected, proper assignment of ancestry also depends on an extensive reference data base. The ancestry inference of the bone remains here was based on a reference population frequency of 3081 individuals from 33 world populations. These populations include ancestries of East Asian, African and European, which allows a better representation of world populations at the three-continent level. Recently, we updated our reference database to a more extensive array of 3826 individuals from 43 worldwide populations [9]. However, this panel cannot achieve better resolution among East Asians. A second-tier AISNP panel and a sub-population reference database should be developed to achieve this goal.

Before performing the 27-plex assay, we would usually conduct STR examinations for personal identification, as well as to confirm if the samples we tested were from a single source or mixtures. During the 27-plex assay procedure, at least three replicates were performed for each test to ensure that the results were reliable. For the current 27-plex assay system, multiple repeated experiments were conducted, and consistent results were obtained, showing that the detection system is stable. However, there was still some imbalance among different fluorochromes and different SNP sites, so we still need to optimize the system.

In conclusion, the 27-plex assay was successfully employed to infer the ancestry of bone remains from the Second World War. This single-tube assay is sensitive enough for common forensic specimens. In addition, it can easily be implemented in most forensic genetic laboratories because the 27-SNP profile is developed/detected using capillary electrophoresis instruments currently used by the majority of forensic laboratories.

## Supplementary Material

fig1.tif
