# Supplementary figures and images for: Twenty-seven continental ancestry-informative SNP analysis of bone remains to resolve a forensic case
Source: Forensic Sci Res. 2017 May 19;4(4):364–6. doi: 10.1080/20961790.2017.1306431 (PMC6968571; doi:10.1080/20961790.2017.1306431)

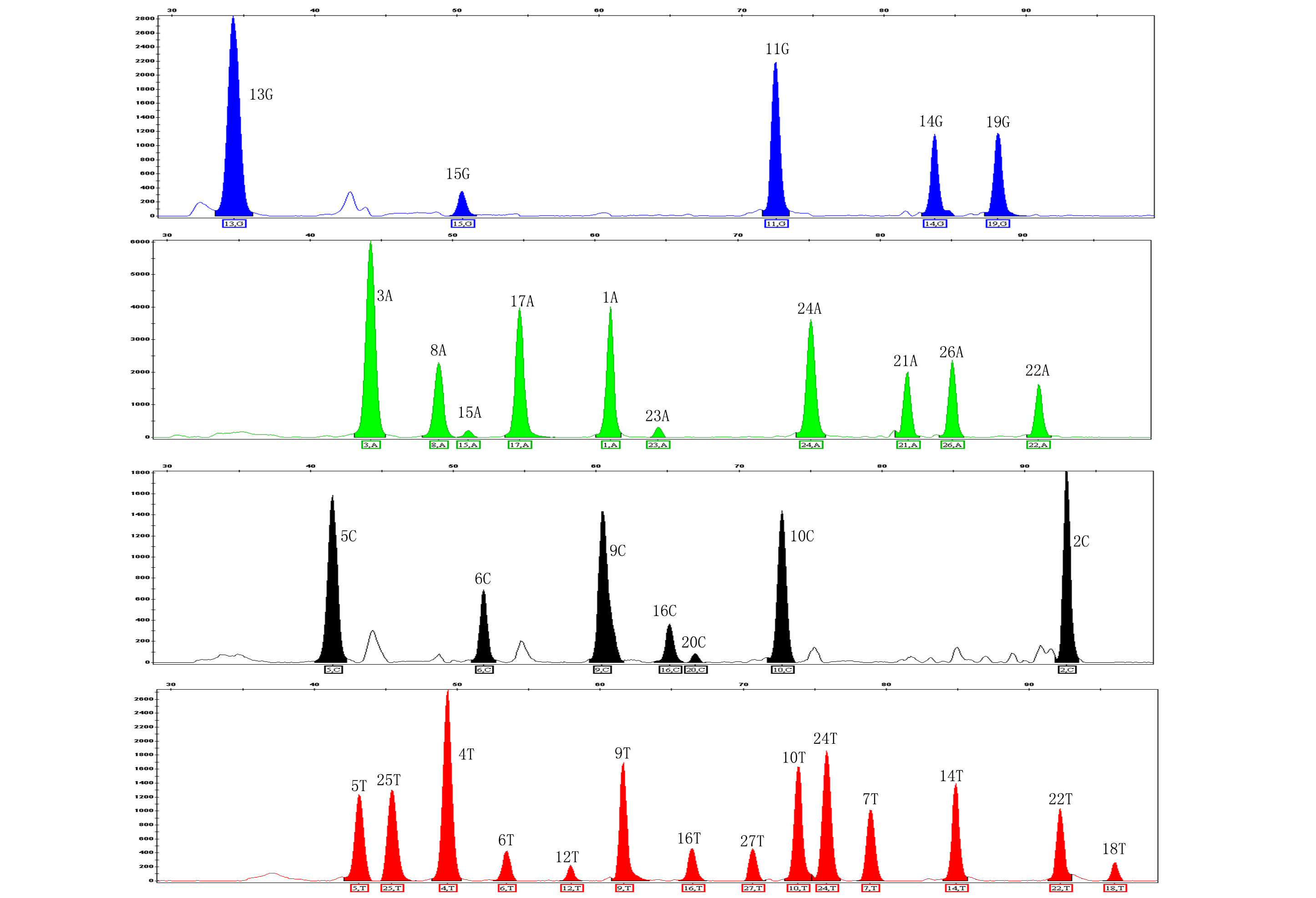

Supplement: fig1.tif [file TFSR_A_1306431_SM4958.tif]
